# Plasma volume expansion across healthy pregnancy: a systematic review and meta-analysis of longitudinal studies

**DOI:** 10.1186/s12884-019-2619-6

**Published:** 2019-12-19

**Authors:** Sixtus Aguree, Alison D. Gernand

**Affiliations:** 110 Chandlee Laboratory, Department of Nutritional Sciences, The Pennsylvania State University, University Park, PA 16802 USA

**Keywords:** Plasma volume expansion, Pregnancy, Gestation, Evans blue dye, Hemodilution

## Abstract

**Background:**

Plasma volume expansion is an important physiologic change across gestation. High or low expansion has been related to adverse pregnancy outcomes, yet there is a limited understanding of normal/healthy plasma volume expansion. We aimed to evaluate the pattern of plasma volume expansion across healthy pregnancies from longitudinal studies.

**Methods:**

We conducted a systematic review and meta-analysis to identify original studies that measured plasma volume in singleton pregnancies of healthy women. Specifically, we included studies that measured plasma volume at least two times across gestation and one time before or after pregnancy in the same women. PubMed, Web of Science, Cochrane, CINAHL, and clinicaltrials.gov databases were searched from the beginning of each database to February 2019. We combined data across studies using a random effects model.

**Results:**

Ten observational studies with a total of 347 pregnancies were eligible. Plasma volume increased by 6% (95% CI 3–9) in the first trimester compared to the nonpregnant state. In the second trimester, plasma volume was increased by 18% (95% CI 12–24) in gestational weeks 14–20 and 29% (95% CI 21–36) in weeks 21–27 above the nonpregnant state. In the third trimester, plasma volume was increased by 42% (95% CI 38–46) in weeks 28–34 and 48% (95% CI 44–51) in weeks 35–38. The highest rate of increase occurred in the first half of the second trimester. Included studies were rated from moderate to high quality; 7 out of 10 studies were conducted over 30 years ago.

**Conclusions:**

In healthy pregnancies, plasma volume begins to expand in the first trimester, has the steepest rate of increase in the second trimester, and peaks late in the third trimester. The patterns observed from these studies may not reflect the current population, partly due to the changes in BMI over the last several decades. Additional longitudinal studies are needed to better characterize the range of normal plasma volume expansion across maternal characteristics.

## Background

Early in the twentieth century, there was emerging evidence that plasma volume increased during pregnancy [1, 2]. Yet, there were conflicting reports about the nature of the expansion, in part because most studies were not longitudinal. In 1934, Dieckmann and Wegner’s formative paper on plasma volume crystalized our understanding that plasma volume increases substantially as part of normal pregnancy [3]. They serially measured plasma volume in the same group of women across pregnancy, and at varying times after pregnancy [3]. As with other physiologic changes during pregnancy that were not well understood or characterized, more knowledge was needed on the range of healthy plasma volume expansion in order to identify abnormal changes that could be part of a disease process.

Since that time, there have been additional studies of plasma volume, but not many. Adverse pregnancy outcomes, including hypertensive disorders of pregnancy, have been linked to reduced plasma volume expansion during pregnancy [4–7]. Plasma volume in the third trimester [8–10] and total plasma volume expansion [10–13] are both positively associated with birthweight. The increase in plasma volume has even been suggested to be more important than maternal stature in terms of its influence on birthweight [10, 14]. As well, plasma volume expansion affects blood-based biomarker concentrations during pregnancy. Hemoglobin concentrations decrease as plasma volume increases to a greater extent than red blood cell mass [13, 15, 16], and diagnostic cut-offs for anemia vary by trimester [17, 18]. However, the relationship between plasma volume and plasma-based biomarker concentrations may be complicated. For instance, higher plasma volume has been associated with lower plasma zinc [19] and folate [20] concentrations but higher plasma copper [19] and ceruloplasmin [19] concentrations. Diagnostic biomarkers could be misinterpreted due to abnormal plasma volume expansion.

Studies describing plasma volume expansion across gestation are limited, in part because measurements are costly and somewhat invasive. Most methods are indicator dilution techniques, requiring a blood draw, injection of a tracer (usually Evans blue dye or indocyanine green), and serial blood collection post-injection. The concentration of the tracer is measured and used to back-extrapolate the concentration of dye at the time of complete mixing, allowing calculation of plasma volume [21]. Methods for measuring plasma volume are reviewed elsewhere [22, 23].

Plasma volume expansion is widely quoted as being 50% above the nonpregnant volume, as if this is a well-established value. The range of normal is rarely mentioned. Most studies report cross-sectional plasma volume during pregnancy, yet one snapshot is inadequate for understanding plasma volume physiology. For example, absolute plasma volume measured at one point during pregnancy may be lower in smaller women compared to women with larger body sizes, but the percentage increase may be higher [11, 14, 24]. A more thorough understanding of the normal pattern of plasma volume expansion in healthy pregnancies could be important for research and clinical care, informing the pathophysiology of disease states and providing data to alert clinicians to poor maternal adaptations ahead of clinical signs of disease.

To our knowledge, longitudinal data on normal plasma volume has not be systematically assessed, leaving a gap in our knowledge of this crucial physiologic change. Our aim was to conduct a systematic review and meta-analysis of longitudinal studies to describe the pattern of plasma volume expansion across gestation in healthy, singleton pregnancies. This review provides an assessment of the amount data available on plasma volume expansion and the characteristics of women in the studies, which can inform next steps in plasma volume research.

## Methods

### Search strategy

We conducted a systematic review and meta-analysis according to the Preferred Reporting Items for Systematic Reviews and Meta-Analyses (PRISMA) guidelines [25]. We identified relevant studies through electronic searches of published literature, together with citation tracking and hand searching of references from published articles. We searched PubMed, Web of Science, Cochrane library and CINAHL databases, and clinicaltrials.gov. In addition, we searched for grey literature sources including ProQuest dissertations and theses. Each database was searched from its beginning to February 2019. We created search strategies in collaboration with a health sciences librarian at our institution with expertise in systematic reviews.

A combination of terms related to plasma volume, blood volume, and pregnancy were used in our search strategy. The Medical Subject Headings (MeSH) of the U.S. National Library of Medicine (NLM) were used whenever possible in PubMed to retrieve articles. The search strategy for PubMed was: (“blood volume” OR “plasma volume” OR “erythrocyte volume” OR “blood volume” [tiab] OR “plasma volume” [tiab] OR “red cell volume” [tiab] OR “erythrocyte volume” [tiab]) AND (“pregnancy” [MeSH] OR “pregnancy” [tiab] OR “gestation” [tiab] OR “gravidity” [MeSH] OR “gravidity” [tiab] OR “gravidities” [tiab] OR “pregnant” [tiab] OR “pregnancies” [tiab] OR “gestational” [tiab]).

The literature search was planned by S.A. and then evaluated by the health sciences librarian. S.A. then used the search strategy to independently search and extract all articles from the databases; results were compared with those from the librarian. The differences were resolved and the final protocol refined until the independent searches produced the same results. The data search was initially conducted from January to June 2017 and was updated in February 2019. All articles were retrieved into an EndNote library and duplicates were removed. Data extraction followed the PRISMA protocol [25]. S.A. extracted the data and both authors checked these for accuracy. Data extraction was limited to full-length published articles.

### Data extraction and quality assessment

We included longitudinal cohort studies that measured plasma volume at least two times across gestation and one time point before or after pregnancy, in community-living (non-hospitalized) healthy pregnant women with singleton gestation. Studies that did not report a measurement before gestational week 35 were excluded because it has been reported that some women reach their peak plasma volume before this time [24, 26]. Studies had to report mean plasma volume with sample size and a standard error (SE), standard deviation (SD), or 95% confidence interval (CI). If a study included both healthy and complicated pregnancies, only the data from subjects with healthy pregnancies were included. Studies were not excluded based on parity, age, race, geographic location, or method of plasma volume measurement. Where multiple published reports from the same population were available, we selected the publication with the most detailed information.

**Fig. 1:**
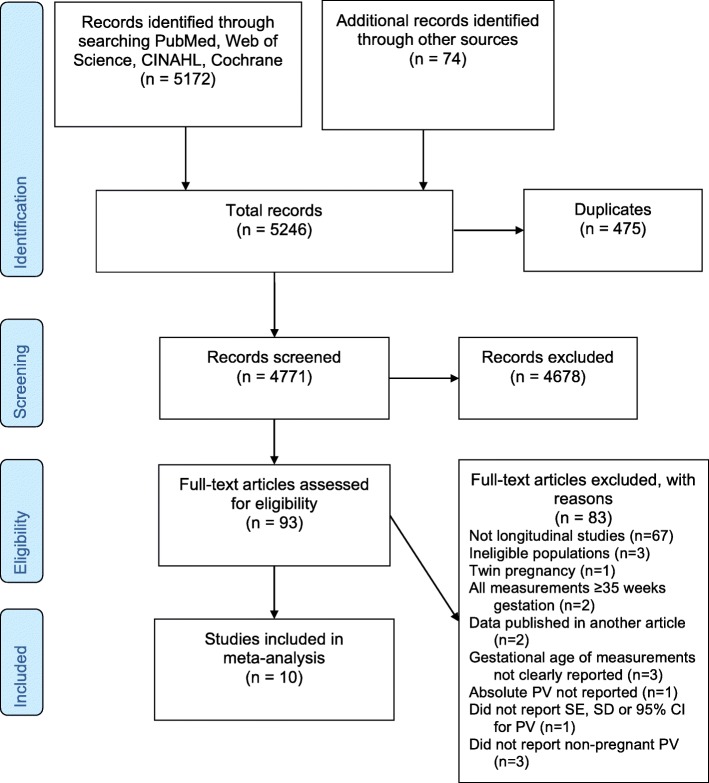
Flow diagram for the systematic review [PRISMA statement [25, 27]]. PRISMA, Preferred Reporting Items for Systematic Reviews and Meta-Analyses; PV, plasma volume

The electronic search resulted in 5246 total titles and abstracts for review (Fig. 1). After removing duplicates and screening titles and abstracts, we reviewed the full-text of 93 studies to assess if eligibility criteria were met. Of note, Chapman et al. examined several systemic and renal hemodynamic measures (including plasma volume) in ten women in the mid follicular phase of the menstrual cycle and weeks 6, 8, 10, 12, 24, and 36 gestations [28]. This was a detailed and well-designed study where plasma volume was reported standardized by weight (mL/kg). The study however did not provide information that would allow conversion of estimates from mL/kg to mL as reported in other studies. It was therefore not possible to include this study in the meta-analysis. Ultimately, 10 studies met all criteria and were included in the final analysis (Fig. 1).

All plasma volume values were converted to mL. Mean plasma volume and SD were recorded by week, including the sample size. When a range of gestational weeks was reported for an individual study’s plasma volume measurement, we used the median gestational week within the interval. Where SD was absent but SE and sample size or mean and 95% CI reported, we estimated SD from those parameters. The outcome variable – mean difference in plasma volume – was calculated by subtracting the nonpregnant value from the corresponding pregnant value. We estimated mean difference from the nonpregnant value for each gestational age interval, and expressed as absolute change and percentage change. Postpartum (≥6 weeks) plasma volume was used as the nonpregnant reference because all studies included this timepoint but only one study included a prepregnancy value [15].

We used the Downs and Black [29] method to assess the quality of the included studies (see Additional file 1). In brief, this involves a 27-item checklist that assesses quality of reporting based on the following areas: reporting, external validity, internal validity, confounding or selection bias, and power. We classified “high quality” similarly to a previous meta-analysis [30].

### Statistical analysis

Weighted mean plasma volume and change in volume were calculated using the random effects model by DerSimonian and Laird, [31] and grouped in five gestational intervals: 7–13, 14–20, 21–27, 28–34, and 35–38 weeks – similar to other studies [32, 33] but modified to have intervals corresponding with conventional trimesters.

**Fig. 2:**
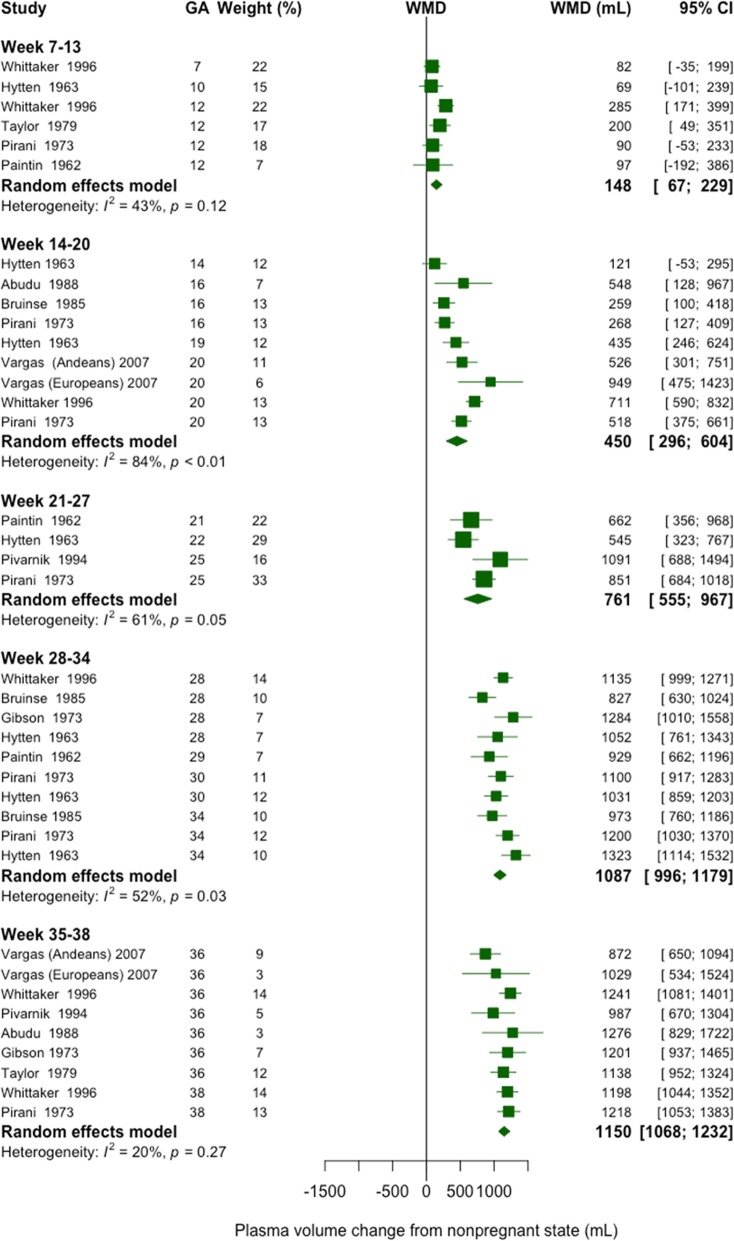
Forest plot displaying effect sizes (plasma volume during pregnancy minus volume after pregnancy) of studies measuring plasma volume across gestation. Analysis conducted with random effects model. GA, gestational age (weeks); WMD, weighted mean difference; CI, confidence interval

**Fig. 3:**
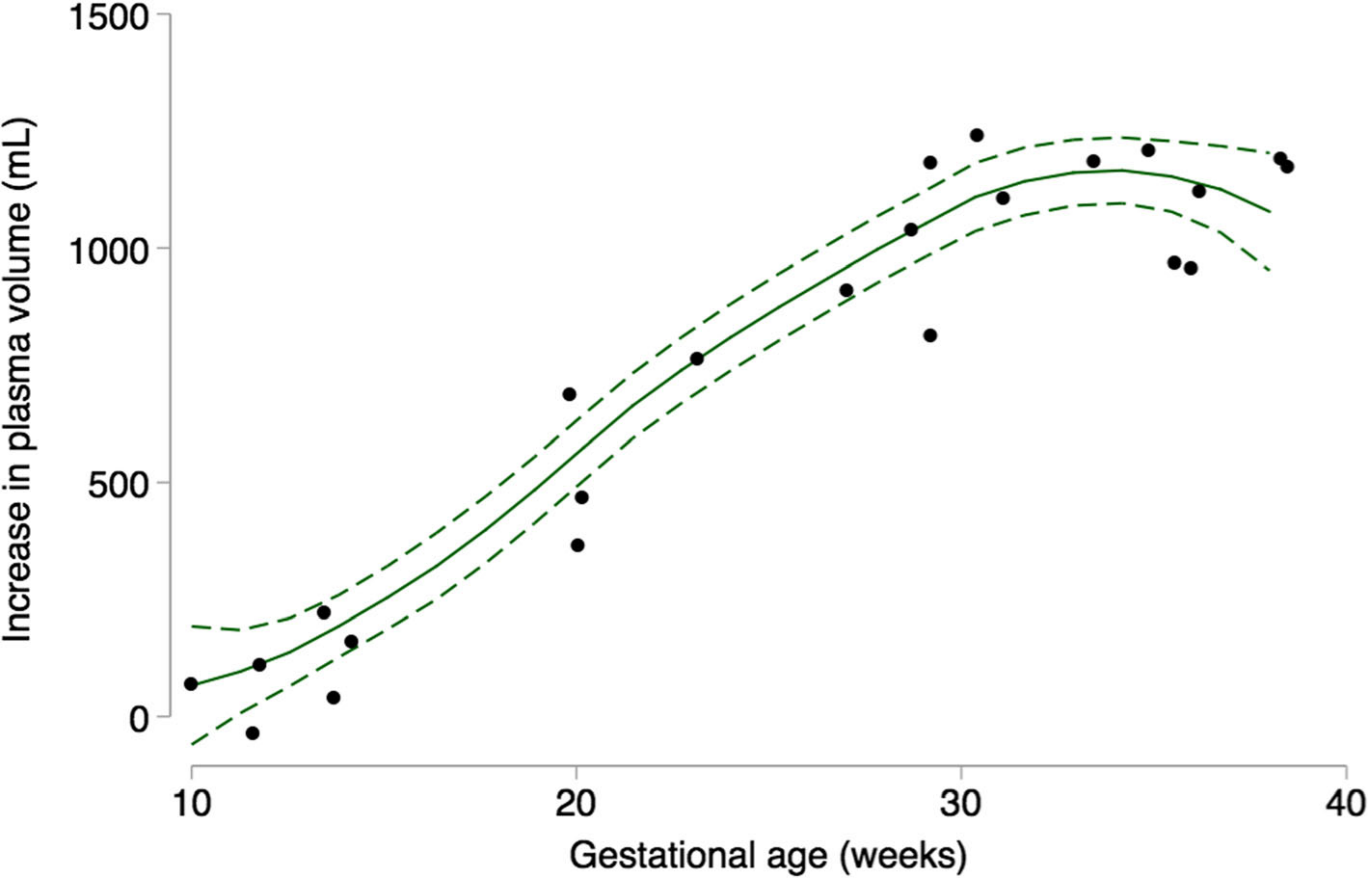
Summary of plasma volume expansion across gestation (*n* = 10 studies; 17 timepoints). Dots represent data from individual studies; solid line represents prediction based on all data; short dashed line represents the 95% CI around the prediction

**Fig. 4:**
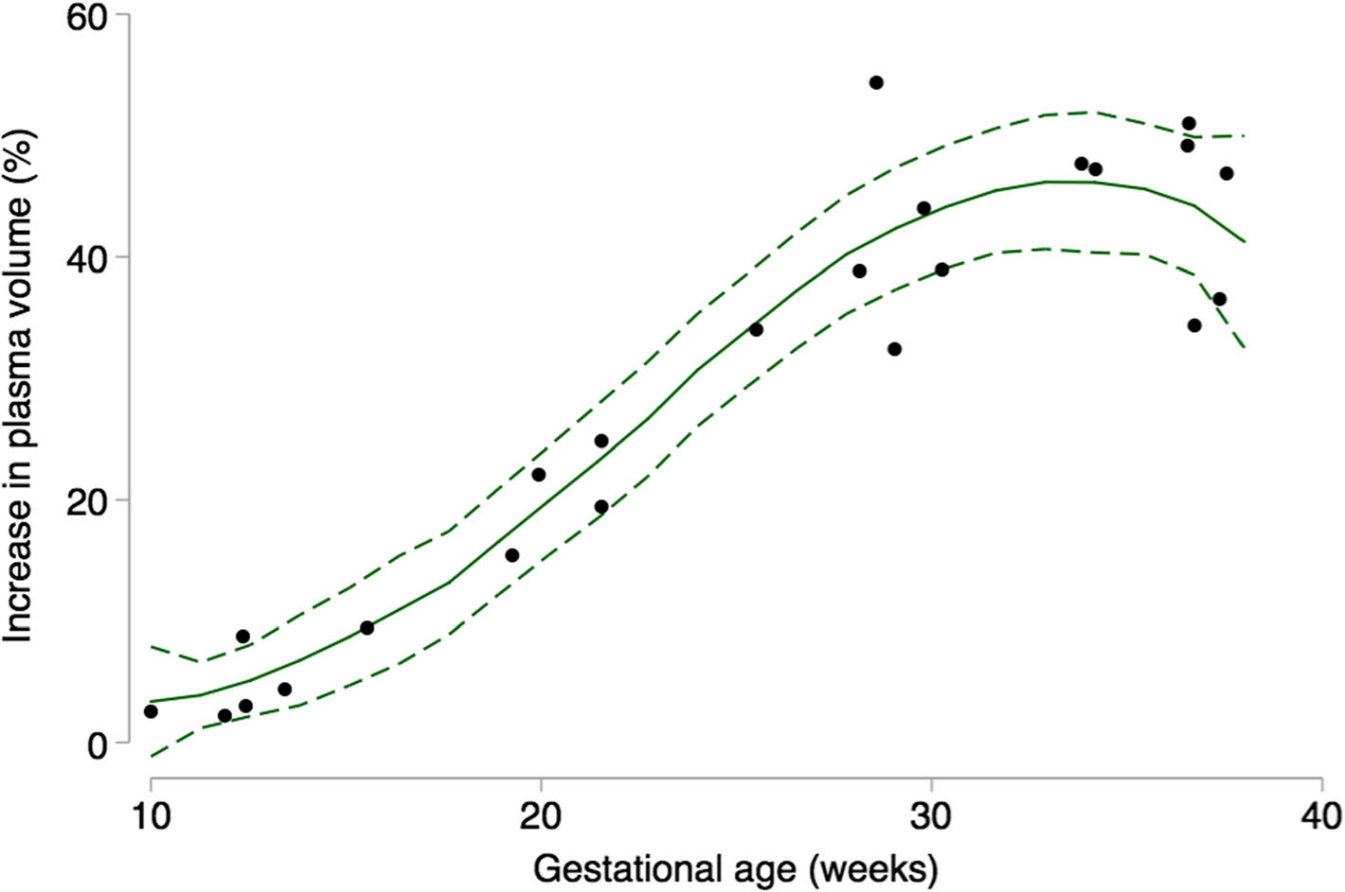
Summary of percent plasma volume expansion across gestation (*n* = 10 studies; 17 timepoints). Dots represent data from individual studies; solid line represents prediction based on all data; short dashed line represents the 95% CI around the prediction

We used Higgins I^2^ to statistically assess heterogeneity [34] and we classified the level of heterogeneity using Higgins et al. suggested cutoffs for I^2^ values: low (25–50%), moderate (50–75%), and high (≥75%) [35]. Publication bias was assessed using a funnel plot, which is a visual assessment of publication bias. Formal statistical testing for publication bias arising from small-study effects was conducted using Egger’s and Begg’s tests [36, 37]. We performed sensitivity analysis by examining the effect of removing one study at a time (leave-one-out analysis) on the pooled estimate at each gestational age interval. We also examined the effect of including the one reported prepregnancy plasma volume as the nonpregnant value [15]. We used the meta package in R version 3.4.3 (R Foundation for Statistical Computing, Vienna, Austria) to estimate weighted mean and weighted mean difference, and to generate Fig. 2 and Supplemental materials (see Additional files 2, 3, 4, and 5) [38–40]. We used a twoway local polynomial smooth plot with 95% CI in Stata version 14 (StataCorp, College Station, Texas) to generate Figs. 3 and 4.

## Results

Ten longitudinal studies including a total of 347 subjects (median sample size 31) were analyzed in the meta-analysis: six from the United Kingdom [10, 13–15, 41, 42], and one study each from the United States of America [43], Bolivia [44], Nigeria [45], and the Netherlands [46] (Table 1). Except for the studies from Nigeria and Bolivia, participants in the other studies were largely white women of European ancestry. Half of the studies were published in the 1960s and 1970s (all from the UK) [10, 13, 14, 41, 42]. The mean maternal age in most studies was < 30 years. Heights were typically within a healthy range, with mean values spanning from 167 cm in the Netherlands to 150 cm in Andeans in Bolivia. Studies reported body weight outside of pregnancy or in early gestation; only one study reported nonpregnant BMI (26.1 ± 0.6 kg/m^2^ for Andeans and 24.7 ± 1.2 kg/m^2^ for Europeans) [44]; it was not possible to examine if the plasma volume expansion pattern for overweight/obese women would be different from women with a healthy weight. Most women were primigravidae.

**Table 1:** Characteristics of studies included in the systematic review and meta-analysis of plasma volume expansion across healthy pregnancy

Author, year	Country	Sample Size	Gravidity	Age (y)	Height (cm)	Weight (kg)	Plasma Volume Method	No. pregnancy measurements	Postpartum^a^	Postpartum plasma volume (mL)
Paintin, 1962 [42]	UK	20	1	18-31^b^	164.4	45.8–78.3^b^	EBD	4	7–8 weeks	2775
Hytten, 1963 [14]	UK	39	1	24.9 ± 5.8	162.4 ± 6.1	58.2 ± 5.9	EBD	8	6–8 weeks	2699
Gibson, 1973 [13]	UK	9	> 1	28.1 ± 2.9	156.7 ± 3.7	56.8 ± 7.3	EBD	2	3 months	2344
Pirani, 1973 [10]	UK	56	1	20.8	155.5	NA	EBD	7	6–8 weeks	2545
Taylor, 1979 [41]	UK	24	1^c^	27.1 ± 3.4	163.5 ± 4.9	60.1 ± 8.8	EBD	2	4–6 months	2340
Bruinse, 1985 [46]	The Netherlands	37	0.6 ± 0.7^d^	25.4 ± 3.6	167.1 ± 5.8	61.7 ± 7.9	EBD	3	6 days, 6 weeks^e^, and 6 months	2557
Abudu, 1988 [45]	Nigeria	20	1	23.9 ± 5.0	163.0 ± 4.1	58.9 ± 7.9	EBD	2	8 weeks	2165
Pivarnik, 1994 [43]	USA	5	NA	29.0 ± 4	159.0 ± 5.0	65.3 ± 4.6	EBD	2	12 weeks	2355
Whittaker, 1996 [15]	UK	69	0–3	29.3^f^	NA	NA	EBD	6	12 weeks^g^	2382
Vargas, 2007 [44]	Bolivia:						CO			
	Andean	42	3.4 ± 0.3	27.2 ± 6.4	150.0 ± 0.6	58.8 ± 1.5		2	4 months	2482
	European	26	2.3 ± 0.2	32.4 ± 4.0	162.0 ± 1.3	63.5 ± 2.5		2	4 months	2631

Abbreviation: *NA* not available *EBD* Evans blue dye, *CO* CO-rebreathing

Data are mean ± SD unless otherwise specified

^a^Time from birth

^b^Range (mean not reported)

^c^Consists of nulliparous, primiparous and multiparous women

^d^Parity reported

^e^Used in the meta-analysis

^f^Age at delivery

^g^Prepregnancy value was also reported (2373 mL)

The total number of repeated measurements during pregnancy ranged from 2 to 8, and study sample sizes ranged from 5 to 68. The timing of postpartum measurements ranged from 8 weeks to about 6 months. Nine of the studies used a postpartum measurement as a proxy for prepregnancy, assuming nonpregnant plasma volume would be similar before and after pregnancy; Whittaker et al. [15] was the only study that reported prepregnancy plasma volume. Nine studies used Evans blue dye and one study used CO-rebreathing [44] to measure plasma volume. Studies were mostly of moderate to high quality.

We performed a meta-analysis of estimated changes in plasma volume (nonpregnant to pregnant) at five discrete gestational age groupings (second and third trimesters were divided into 2 groups each; Fig. 2). Overall, the heterogeneity was acceptable, and studies were combined. The increase in mean plasma volume at the end of the first trimester was 6% (95% CI 3–9). In the second trimester, the increase above the nonpregnant state was 18% (95% CI 12–24) in gestational weeks 14–20, and 29% (95% CI 21–36) in weeks 21–27. In the third trimester, the increase was 42% (95% CI 39–46) in weeks 28–34, and 48% (95% CI 44–51) in weeks 35–38.

We then assessed the rate of change between weighted means at each interval. The increase in plasma volume from the first trimester to the first half of the second trimester was 302 mL (a threefold increase in the gain, 148 vs. 450 mL). There was a statistically significant increase (*P* < 0.05) in plasma volume from each gestational interval to the next except for weeks 28–34 vs. 35–38 (1087 vs. 1150 mL). The highest rate of volume increase was seen in the second trimester. Plasma volume continued to increase in the latter half of the second trimester and third trimester, but the rate of increase was lower. We had similar findings for all meta-analyses when we used the prepregnant mean plasma volume instead of the post-partum value from the Whittaker et al. study [15], and when we excluded the one study that measured plasma volume with CO-rebreathing.

We also examined data from each of the ten individual studies. The results are presented in terms of absolute change (mL; Fig. 3) and relative change (%; Fig. 4) from the nonpregnant state reported in each study. The earliest gestational age reported was at week seven (one study, 3% increase) [15], and the latest was week 38 (two studies: 48 and 50% increases [10, 15]; see Additional file 2). The largest percentage increase was 59% at week 36 [45], while the largest volume increase was 1323 mL at 34 weeks [14]. There was a steady increase in plasma volume in the first trimester, followed by a steep rise between weeks 12 and 30, then a slower increase to term (Figs. 3 and 4). The mean nonpregnant plasma volume was 2529 mL across all studies. Overall, plasma volume increased in each trimester, with the largest difference in volume occurring between the first and second trimesters, for each study. However, two studies reported a lower plasma volume in the third compared to second trimester [13, 43]. Only one study of first pregnancies reported the range of expansion at peak volume (25–80%, 630–1940 mL), yet the gestational age at peak was not specified [14].

Heterogeneity (I^2^) between studies was low in the first and third trimesters, and moderate to high in the second trimester (Fig. 2). Heterogeneity was lower when we stratified studies into smaller, monthly gestational intervals (see Additional file 4). There was no evidence of publication bias from visual inspection of funnel plots for each gestational interval (see Additional file 5). Both Egger’s test (0.367 ≤ *P* ≤ 0.883) and Begg’s test (0.312 ≤ *P* ≤ 0.851) showed no evidence of small-study effects. Results from the leave-one-out analysis did not show evidence of extreme influence from any one particular study. The sensitivity analysis showed that none of the studies had a large effect on the pooled estimates, at any gestational interval (see Additional file 3).

## Discussion

The current paper reviewed and synthesized studies with repeated measures of plasma volume from the same group of women with healthy pregnancies. Overall, 10 observational, longitudinal studies (347 total women) examining plasma volume changes across gestation in comparison to nonpregnant values were included in the meta-analysis. Based on this limited data, plasma volume increased to a small but measureable extent in the first trimester, followed by a sharp rise in the second trimester and a continuous but slow increase in the third trimester. The maximum weighted mean increase was 48% (1150 mL) above the nonpregnant volume, yet mean increases (percentage and/or volume) and the gestational age at peak expansion varied between studies.

Hytten was dedicated to understanding changes during normal pregnancy and reviewed studies of plasma volume in several publications including in his book “The Physiology of Human Pregnancy” [47] and a later review paper in Clinics in Haematology [48]. He did not use formal meta-analysis, but in compiling the few available studies, concluded that plasma volume expanded by just under 50% (1250 mL) among healthy women of European decent [48]. The range of normal plasma volume expansion was not provided for the first and second trimesters, nor the range of expansion around the 50% estimate. He acknowledged many factors that appear to influence baseline plasma volume and expansion during pregnancy, including maternal size, parity, and multiple gestation; these factors have been documented by Hytten and others [49]. While the available longitudinal data did not allow for stratification by these maternal factors, our meta-analysis estimate of a 48% maximum expansion was similar to Hytten’s estimate over 30 years ago.

Since the 1980s, there has continued to be a small amount of research on plasma volume. A recent review and meta-analysis combined studies from 1934 to 2007 to estimate plasma volume at gestational intervals across pregnancy compared to nonpregnancy [33]. Particularly informative, the authors compared plasma volume changes between healthy pregnancies and pregnancies with adverse outcomes such as preeclampsia, and found that expansion was lower in pregnancies with poor outcomes compared to healthy outcomes (32% vs. 46%). This was a comprehensive analysis, but unlike the current review, cross-sectional studies were included. Two concerns arise in using cross-sectional data to establish normal plasma volume expansion: 1) values during pregnancy and outside of pregnancy may be quite different for different women (see factors noted above) and 2) expansion is a change within an individual which inherently requires longitudinal data. Nevertheless, our estimates of maximum plasma volume expansion and the pattern of plasma volume expansion are similar to the expansion and pattern reported for healthy women in the de Haas et al. meta-analysis [33], showing consistency of findings across study designs.

Most studies reporting plasma volume expansion during pregnancy rely on a small number of women, few measurements, and minimal data reporting, making it difficult to know what is normal for aspects such as the range of volumes at peak expansion, the range of gestational ages at peak expansion, and the pattern of change beginning from periconception. Furthermore, we know even less about how these factors may differ across maternal characteristics such as weight, parity, age, and race/ethnicity. Still largely unknown is the degree of variability across different women with healthy pregnancies. The range of normal plasma volume expansion in healthy pregnancies has been reported as 25 to 80% [14] and 43 to 78% [50], but in general is rarely provided in publications. There may be a wide range in the gestational age at which women reach peak plasma volume (and whether or not it is maintained or slightly declines until delivery), and some studies have reported that women can reach peak volume as early as the second trimester [13, 43, 51]. Many aspects of volume and expansion are not uniformly reported in studies. The physiological mechanism responsible for plasma volume expansion during pregnancy are reviewed elsewhere [52, 53]. Briefly, it is thought that the activation of the renin-angiotensin-aldosterone system drives the rise in plasma volume during pregnancy.

Knowledge of the normal trend in plasma volume expansion, and the ability to measure it in clinical settings, is important because plasma volume expansion is a critical change in pregnancy needed for blood flow to the uterus, and it is associated with many health conditions. Low plasma volume is associated with an increased risk of developing gestational hypertension compared to normal plasma volume [4]. Furthermore, low pre-pregnancy plasma volume has been associated with recurrent preeclampsia, recurrent pregnancy loss, and risk of preterm delivery [5, 54]. In research, detailed data on plasma volume could inform the pathophysiology of preeclampsia, fetal growth restriction, and other adverse outcomes. In clinical care, if reference curves and a simple method to measure plasma volume were developed, abnormal expansion in early pregnancy might predict disease progression before clinical onset, allowing earlier opportunities for clinical intervention.

Plasma volume may also affect biomarker concentrations. Biomarkers are important for clinical care, public health surveillance, and research alike. Some nutritional status biomarkers like vitamin B6, folate, zinc, copper, and hemoglobin have all been shown to change across gestation; the role of plasma volume in these changes has not been well described except for hemoglobin (creating lower cutoffs to diagnose anemia in pregnancy) [17, 55]. It is important to understand what level of changes in biomarkers are physiological and at what level of change should intervention be given to improve maternal and birth outcomes. Concurrently measuring plasma volume and nutritional biomarkers may be needed to understand these relationships.

Most of the studies available for this review were done in the 1960s and 1970s, a period when pregnant women were generally younger and leaner than those today [56–61]. It is unclear if the same pattern of expansion would be observed for older pregnant women [62], and maternal age during pregnancy has been continuing to rise in the US [63, 64]. Similarly, the prevalence of prepregnancy overweight and obesity has increased dramatically since the 1990s [56, 57, 59, 60]. It is likely that BMI could impact plasma volume [65], but the available studies report raw weight not BMI. On the other hand, this study included only healthy women, so the findings observed may be a good representation of healthy plasma volume expansion for comparisons (e.g., if data on plasma volume in women with obesity are collected). There is also a strong interest in the full range of normal plasma volume, not just the mean, but this was only reported for the peak plasma volume expansion in one study [14].

We considered several sources of bias as part of our review. Individual studies likely had a low risk of selection bias, a higher concern when trying to study all pregnant women or specific adverse outcomes, and confounding, as no exposure-outcome relationships were examined. As studies had relatively high quality scores in these areas (Additional file 1), we do not expect these sources of bias to have influenced our findings. On the other hand, assessing the potential for information bias was challenging. While studies were small and we expect the measurement of plasma volume was carefully conducted and recorded, body position during measurements could have been a source of error, particularly late in pregnancy. Body position is important for measurements and there were several different positions (e.g., lying on side, sitting reclined) employed in the studies, or it was not clearly reported. Hytten previously described this issue and noted that measurements from women lying supine were lower near term compared to women lying on one side, due to the weight of the uterus compressing the inferior vena cava and resulting in incomplete mixing of the dye [48]. Though our leave-one-out analysis showed that no individual study had a major impact on results (Additional file 3), we cannot rule out the possibility that errors in measurement, particularly late in gestation for women not lying on one side, did not result in lower plasma volume values. Future work should standardize the position to be lateral during measurements. Overall, plasma volume estimates varied considerably as reflected in the high heterogeneity in studies, particularly during the second trimester. Though the funnel plot did not suggest publication bias, it is difficult to know if there were no other sources of reporting bias because of the small number of studies included [66].

A strength and unique aspect of this review was that we included only longitudinal studies, which had as many as eight repeated measurements during pregnancy and which always measured nonpregnant plasma volume in the same cohort of women. As well, 17 separate gestational time points in pregnancy were represented and plasma volume was always measured by “direct” methods, rather than indirect methods such as calculations based on hemoglobin and hematocrit changes. We assessed numerous aspects of the available data, including the maximum change, the change up to discrete gestational intervals, the rate of change between gestational intervals, and important aspects from individual studies.

## Conclusions

This meta-analysis suggests that plasma volume increases by 6% in the first trimester, by 29% at the end of the second trimester, and 48% (peak expansion) near term, based on data from 347 healthy women with uncomplicated pregnancies – confirming previous estimates of a 46–50% peak expansion. Our knowledge of plasma volume expansion in pregnancy is based on a limited number of women, mostly white, normal weight women, and relatively young participants. Most studies in this area were conducted 50 to 60 years ago, at the time obesity prevalence was low, and women generally entered pregnancy at a much younger age.

Future studies should be longitudinal and conducted in diverse groups of women, including prepregnancy measurements (with standardization for menstrual cycle phase). Work is needed to develop and test new methods for reliable, and non-invasive, plasma volume measurements during pregnancy, especially because Evans Blue Dye is no longer available in many countries due to safety concerns. Compared to gestational weight gain, where healthy weight gain is based on rigorous studies involving thousands of participants, we do not have large numbers of plasma volume studies to draw conclusions regarding normal expansion. Large datasets would be useful to create reference values for plasma volume expansion across weeks of gestation, and in turn, epidemiologic surveillance could track changes in plasma volume expansion and associations with other outcomes such as maternal obesity and SGA. Additional recommendations for future work include examining the effect of plasma volume expansion on plasma-based biomarkers. Baseline knowledge about plasma volume in pregnancy has been established, but our knowledge of this important physiologic change in pregnancy needs to greatly expand.

## Supplementary information

**Additional file 1.** Quality score for each study by area of threat to validity and overall.

**Additional file 2.** Plasma volume expansion for individual studies.

**Additional file 3.** Leave-one-out sensitivity analysis.

**Additional file 4.** Forest plot of plasma volume expansion by four-week intervals. WMD, weighted mean difference; GA, gestational age (weeks); WMD, weighted mean difference; CI, confidence interval.

**Additional file 5.** Funnel plot from random effects meta-analysis of 10 studies. Small green cycles indicate point estimates for included studies. Dotted green inner lines indicate summary WMD. Dotted black outer lines indicate pseudo 95% CI; SE, standard error; WMD, weighted mean difference; Panel A, gestation week 7–13; Panel B, gestation week 14–20; Panel C, gestation week 21–28; Panel D, gestation week 28–34; Panel E, gestation week 35–38.

## Data Availability

All data pertaining to this study are included in this article and supplementary files, or in the original published articles.

## Abbreviations

MeSH: Medical Subject Headings
NLM: National Library of Medicine
PRISMA: Preferred Reporting Items for Systematic Reviews and Meta-Analyses
