# Supplementary material for: Plasma volume expansion across healthy pregnancy: a systematic review and meta-analysis of longitudinal studies
Source: BMC Pregnancy Childbirth. 2019 Dec 19;19:508. doi: 10.1186/s12884-019-2619-6 (PMC6924087; doi:10.1186/s12884-019-2619-6)
Supplement: Supplementary file 1 — Additional file 1. Quality score for each study by area of threat to validity and overall. [file 12884_2019_2619_MOESM1_ESM.doc]

**SUPPLEMENTAL TABLE 2** Quality scores for each study by area of threat to validity and overall [28]

|  | Area of Threat to Validity (Possible Points) | | | | |  |
| --- | --- | --- | --- | --- | --- | --- |
| First Author, Year | Reporting (10) | External Validity (3) | Internal Validity (7) | Confounding or Selection Bias (6) | Power (1) | Total (27) |
|  | Points Scored | | | | |  |
| Paintin, 1962 [41] | 8^*^ | 1 | 5 | 4 | 0 | 18 |
| Hytten, 1963 [14] | 9^*^ | 2 | 5 | 4 | 1^*^ | 21 |
| Gibson, 1973 [13] | 6 | 1 | 4 | 4 | 0 | 15 |
| Pirani, 1973 [10] | 10^*^ | 3^*^ | 6^*^ | 5^*^ | 1^*^ | 25^†^ |
| Taylor, 1979 [40] | 9^*^ | 3^*^ | 6^*^ | 4 | 0 | 22 |
| Bruinse, 1985 [45] | 9^*^ | 2 | 6^*^ | 4 | 1^*^ | 22 |
| Abudu, 1988 [44] | 9^*^ | 2 | 5 | 4 | 0 | 20 |
| Pivarnik, 1994 [42] | 10^*^ | 2 | 5 | 4 | 0 | 21 |
| Whittaker, 1996 [15] | 10^*^ | 2 | 6^*^ | 5^*^ | 1^*^ | 24^†^ |
| Vargas, 2007 [43] | 10^*^ | 2 | 6^*^ | 4 | 1^*^ | 23 |

*Considered high quality within given area of threat to validity (received ≥80% of points).

†Considered high quality study overall (rated high quality in at least four of the five areas of threat to validity).
