# Supplementary material for: Plasma volume expansion across healthy pregnancy: a systematic review and meta-analysis of longitudinal studies
Source: BMC Pregnancy Childbirth. 2019 Dec 19;19:508. doi: 10.1186/s12884-019-2619-6 (PMC6924087; doi:10.1186/s12884-019-2619-6)
Supplement: Supplementary file 2 — Additional file 2. Plasma volume expansion for individual studies. [file 12884_2019_2619_MOESM2_ESM.doc]

**SUPPLEMENTAL TABLE 3** Plasma volume expansion for individual studies

|  | **GA** | **Mean Difference^a^** | |
| --- | --- | --- | --- |
| **Study** | (Weeks) | **mL** | **%** |
| Whittaker 1996 | 7 | 82 | 3 |
| Hytten 1963 | 10 | 69 | 3 |
| Whittaker 1996 | 12 | 285 | 12 |
| Taylor 1979 | 12 | 200 | 9 |
| Pirani 1973 | 12 | 90 | 4 |
| Paintin 1962 | 12 | 97 | 3 |
| Hytten 1963 | 14 | 121 | 4 |
| Abudu 1988 | 16 | 548 | 25 |
| Bruinse 1985 | 16 | 259 | 10 |
| Pirani 1973 | 16 | 268 | 11 |
| Hytten 1963 | 19 | 435 | 16 |
| Vargas 2007 Andeans | 20 | 526 | 21 |
| Vargas 2007 Europeans | 20 | 949 | 36 |
| Whittaker 1996 | 20 | 711 | 30 |
| Pirani 1973 | 20 | 518 | 20 |
| Paintin 1962 | 21 | 662 | 24 |
| Hytten 1963 | 22 | 545 | 20 |
| Pivarnik 1994 | 25 | 1091 | 46 |
| Pirani 1973 | 25 | 851 | 33 |
| Whittaker 1996 | 28 | 1135 | 48 |
| Bruinse 1985 | 28 | 827 | 32 |
| Gibson 1973 | 28 | 1284 | 55 |
| Hytten 1963 | 28 | 1052 | 39 |
| Paintin 1962 | 29 | 929 | 33 |
| Pirani 1973 | 30 | 1100 | 43 |
| Hytten 1963 | 30 | 1031 | 38 |
| Bruinse 1985 | 34 | 973 | 38 |
| Pirani 1973 | 34 | 1200 | 47 |
| Hytten 1963 | 34 | 1323 | 49 |
| Vargas 2007 Andeans | 36 | 872 | 35 |
| Vargas 2007 Europeans | 36 | 1029 | 39 |
| Whittaker 1996 | 36 | 1241 | 52 |
| Pivarnik 1994 | 36 | 987 | 42 |
| Abudu 1988 | 36 | 1276 | 59 |
| Gibson 1973 | 36 | 1201 | 51 |
| Taylor 1979 | 36 | 1138 | 49 |
| Whittaker 1996 | 38 | 1198 | 50 |
| Pirani 1973 | 38 | 1218 | 48 |

Abbreviation: GA, gestational age.

^a^Mean volume at each gestational age minus mean nonpregnant value within each study.
