# Supplementary material for: Plasma volume expansion across healthy pregnancy: a systematic review and meta-analysis of longitudinal studies
Source: BMC Pregnancy Childbirth. 2019 Dec 19;19:508. doi: 10.1186/s12884-019-2619-6 (PMC6924087; doi:10.1186/s12884-019-2619-6)
Supplement: Supplementary file 3 — Additional file 3. Leave-one-out sensitivity analysis. [file 12884_2019_2619_MOESM3_ESM.doc]

**SUPPLEMENTAL TABLE 4 Leave-one-out sensitivity analysis**

| **Study Omitted** | **GA**  **(weeks)** | **Mean Difference** | **95% CI** |
| --- | --- | --- | --- |
| **Weeks 7-13** |  |  |  |
| Whittaker 1996 | 7 | 166 | 73; 259 |
| Hytten 1963 | 10 | 161 | 70; 252 |
| Whittaker 1996 | 12 | 107 | 38; 176 |
| Taylor 1979 | 12 | 135 | 38; 233 |
| Pirani 1973 | 12 | 159 | 64; 255 |
| Paintin 1962 | 12 | 151 | 61; 240 |
| *Pooled estimate* |  | 148 | 61; 240 |
| **Weeks 14-20** |  |  |  |
| Hytten 1963 | 14 | 492 | 343; 640 |
| Abudu 1988 | 16 | 443 | 280; 605 |
| Bruinse 1985 | 16 | 478 | 310; 646 |
| Pirani 1973 | 16 | 477 | 309; 646 |
| Hytten 1963 | 19 | 454 | 280; 628 |
| Vargas (Andeans) 2007 | 20 | 442 | 272; 612 |
| Vargas (Europeans) 2007 | 20 | 417 | 263; 571 |
| Whittaker 1996 | 20 | 397 | 264; 530 |
| Pirani 1973 | 20 | 443 | 264; 622 |
| *Pooled estimate* |  | 450 | 296; 604 |
| **Week 21-27** |  |  |  |
| Paintin 1962 | 21 | 796 | 525; 1067 |
| Hytten 1963 | 22 | 842 | 659; 1025 |
| Pivarnik 1994 | 25 | 700 | 497; 903 |
| Pirani 1973 | 25 | 727 | 438; 1015 |
| *Pooled estimate* |  | 761 | 555; 967 |
| **Week 28-34** |  |  |  |
| Whittaker 1996 | 28 | 1079 | 973; 1186 |
| Bruinse 1985 | 28 | 1118 | 1041; 1197 |
| Gibson 1973 | 28 | 1072 | 978; 1166 |
| Hytten 1963 | 28 | 1090 | 991; 1188 |
| Paintin 1962 | 29 | 1100 | 1004; 1196 |
| Pirani 1973 | 30 | 1085 | 981; 1189 |
| Hytten 1963 | 30 | 1094 | 991; 1197 |
| Bruinse 1985 | 34 | 1099 | 1001; 1198 |
| Pirani 1973 | 34 | 1072 | 972; 1172 |
| Hytten 1963 | 34 | 1063 | 976; 1149 |
| *Pooled estimate* |  | 1087 | 996; 1179 |

**SUPPLEMENTAL TABLE 4 Leave-one-out sensitivity analysis (cont.)**

| **Study omitted** | **GA**  **(weeks)** | **Mean Difference** | **95% CI** |
| --- | --- | --- | --- |
| **Week 35-38** |  |  |  |
| Vargas (Andeans) 2007 | 36 | 1189 | 1114; 1263 |
| Vargas (Europeans) 2007 | 36 | 1152 | 1064; 1239 |
| Whittaker 1996 | 36 | 1130 | 1038; 1221 |
| Pivarnik 1994 | 36 | 1161 | 1076; 1245 |
| Abudu 1988 | 36 | 1144 | 1056; 1231 |
| Gibson 1973 | 36 | 1142 | 1050; 1234 |
| Taylor 1979 | 36 | 1148 | 1051; 1245 |
| Whittaker 1996 | 38 | 1136 | 1037; 1234 |
| Pirani 1973 | 38 | 1134 | 1038; 1229 |
| *Pooled estimate* |  | 1150 | 1068; 1232 |

Abbreviation: GA, gestational age.
