# Supplementary figures and images for: Plasma volume expansion across healthy pregnancy: a systematic review and meta-analysis of longitudinal studies
Source: BMC Pregnancy Childbirth. 2019 Dec 19;19:508. doi: 10.1186/s12884-019-2619-6 (PMC6924087; doi:10.1186/s12884-019-2619-6)

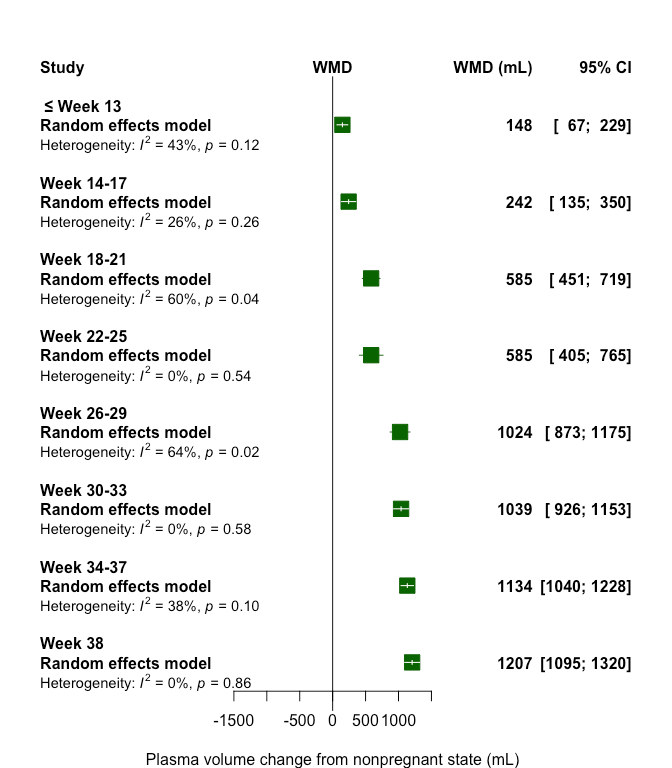

Supplement: Supplementary file 4 — Additional file 4. Forest plot of plasma volume expansion by four-week intervals. WMD, weighted mean difference; GA, gestational age (weeks); WMD, weighted mean difference; CI, confidence interval. [file 12884_2019_2619_MOESM4_ESM.tif]

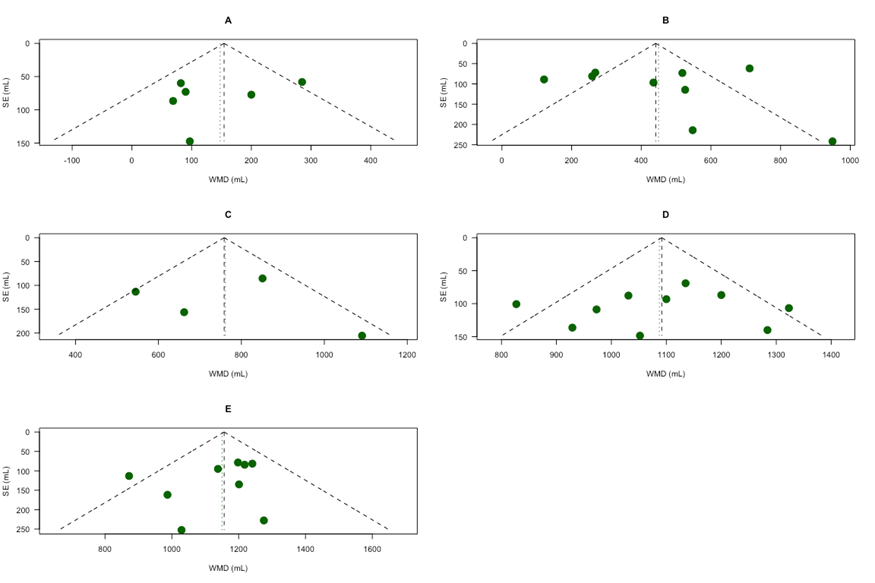

Supplement: Supplementary file 5 — Additional file 5. Funnel plot from random effects meta-analysis of 10 studies. Small green cycles indicate point estimates for included studies. Dotted green inner lines indicate summary WMD. Dotted black outer lines indicate pseudo 95% CI; SE, standard error; WMD, weighted mean difference; Panel A, gestation week 7–13; Panel B, gestation week 14–20; Panel C, gestation week 21–28; Panel D, gestation week 28–34; Panel E, gestation week 35–38. [file 12884_2019_2619_MOESM5_ESM.tif]
